# ChREBP-Knockout Mice Show Sucrose Intolerance and Fructose Malabsorption

**DOI:** 10.3390/nu10030340

**Published:** 2018-03-10

**Authors:** Takehiro Kato, Katsumi Iizuka, Ken Takao, Yukio Horikawa, Tadahiro Kitamura, Jun Takeda

**Affiliations:** 1 Department of Diabetes and Endocrinology, Graduate School of Medicine, Gifu University, Gifu 501-1194, Japan; bado_aberu@yahoo.co.jp (T.K.); lamgerrpard@yahoo.co.jp (K.T.); yhorikaw@gifu-u.ac.jp (Y.H.); jtakeda@gifu-u.ac.jp (J.T.); 2 Gifu University Hospital Center for Nutritional Support and Infection Control, Gifu 501-1194, Japan; 3 Metabolic Signal Research Center, Institute for Molecular and Cellular Regulation, Gunma University, Gunma 371-8512, Japan; kitamura@gunma-u.ac.jp

**Keywords:** carbohydrate-responsive element-binding protein, ketohexokinase, fructose, glucose transporter 5, glucose transporter 2

## Abstract

We have previously reported that 60% sucrose diet-fed *ChREBP* knockout mice (KO) showed body weight loss resulting in lethality. We aimed to elucidate whether sucrose and fructose metabolism are impaired in KO. Wild-type mice (WT) and KO were fed a diet containing 30% sucrose with/without 0.08% miglitol, an α-glucosidase inhibitor, and these effects on phenotypes were tested. Furthermore, we compared metabolic changes of oral and peritoneal fructose injection. A thirty percent sucrose diet feeding did not affect phenotypes in KO. However, miglitol induced lethality in 30% sucrose-fed KO. Thirty percent sucrose plus miglitol diet-fed KO showed increased cecal contents, increased fecal lactate contents, increased growth of lactobacillales and *Bifidobacterium* and decreased growth of clostridium cluster XIVa. *ChREBP* gene deletion suppressed the mRNA levels of sucrose and fructose related genes. Next, oral fructose injection did not affect plasma glucose levels and liver fructose contents; however, intestinal sucrose and fructose related mRNA levels were increased only in WT. In contrast, peritoneal fructose injection increased plasma glucose levels in both mice; however, the hepatic fructose content in KO was much higher owing to decreased hepatic *Khk* mRNA expression. Taken together, KO showed sucrose intolerance and fructose malabsorption owing to decreased gene expression.

## 1. Introduction

Excess intake of high sucrose and fructose diet were thought to be associated with the development of obesity, metabolic syndrome, and diabetes [1,2]. Many experimental animal studies, for example, experiments feeding 70% fructose-containing water, supported this hypothesis [2]. However, recent human epidemic data suggest that there is little association between metabolic syndrome and consumption of sucrose and fructose [3,4]. 

Moreover, the mechanism of sucrose and fructose metabolism remains unclear. Sucrose is a disaccharide composed of glucose and fructose, and is digested by intestinal sucrase-isomaltase (SI), which is inhibited by miglitol, an α-glucosidase inhibitor [5]. Fructose is more potent and has higher capacity of protein glycation than glucose and, thus, is more harmful than glucose [6]. Fructose is metabolized in the intestine and liver. Previously, it has been considered that large amounts of fructose are metabolized mainly in the liver [7]. However, portal fructose levels are five times lower and plasma fructose levels are 100 times lower than plasma glucose levels [8,9]. Moreover, excess intake of fructose can cause dietary fructose malabsorption and thereby irritable bowel syndrome [10]. Taken together, we hypothesized that intestinal fructose absorption, but not hepatic fructose metabolism, regulates portal and plasma fructose levels [11]. 

To clarify the intestinal sucrose and fructose metabolism, we focused on the phenotypes of high-sucrose diet-fed carbohydrate-responsive element-binding protein (ChREBP)-knockout (KO) mice [12]. ChREBP is a glucose-activated transcription factor that regulates glucose and lipid metabolism. We have formerly reported that high-sucrose diet-fed KO mice showed body weight loss and eventual lethality, although high-glucose diet-fed and high-starch diet-fed KO mice did not [12]. As SI is induced by sucrose, we wondered whether SI expression is decreased in KO mice [13]. Moreover, high-fructose diet-fed KO mice showed similar phenotypes (body weight loss and appetite loss) [14,15,16]. ChREBP regulates the gene expression of glucose transporter 5 (*Glut5*) and ketohexokinase (*Khk*), which regulate fructolysis [12,17,18]. Taken together, we speculated that altered sucrose and fructose metabolism may contribute to the pathology of sucrose intolerance and fructose malabsorption seen in KO mice.

In this study, we focused on the effect of ChREBP on sucrose and fructose metabolism in the liver and intestine. We tested whether 30% sucrose plus miglitol (S + M) diet-fed KO mice show phenotypes similar to sucrose intolerance. Furthermore, by comparing the results of oral and peritoneal fructose injection, we tried to clarify the role of hepatic and intestinal ChREBP in fructose metabolism. This study will be beneficial for understanding the mechanism of sucrose and fructose metabolism.

## 2. Materials and Methods 

### 2.1. Materials

Sucrose, fructose, and glucose measurement kits were purchased from Wako Pure Chemical Industries (Osaka, Japan). Lactate measurement kits were purchased from Kyowa Medex Co. (Tokyo, Japan). Triglyceride and cholesterol measurement kits were purchased from Wako Pure Chemical Industries. Glucose-6-phosphate dehydrogenase (G6PDH), phosphoglucose isomerase, hexokinase, and NADP were purchased from Roche Custom Biotech Inc. (Mannheim, Germany).

### 2.2. Animals, and Sucrose and Sucrose + Miglitol Diets

Animal experiments were carried out in accordance with the National Institutes of Health guide for the care and use of Laboratory animals (NIH Publications No. 8023, revised 1978). All animal care was approved by the Animal Care Committee of the University of Gifu (Approval number 27–31, Approval date 4 June 2015). Mice were housed at 23 °C on a 12-h light/dark cycle. KO mice were backcrossed for at least 10 generations onto the C57BL/6J background [19]. 

Mice had free access to water and were fed an autoclaved CE-2 diet (CLEA Japan, Tokyo, Japan). Wild-type (WT) and KO mice were housed separately with a total of three mice per cage. To examine mortality and body weight changes, 12 week old male WT and KO mice were fed a 30% sucrose diet (S; protein 17% kcal, carbohydrate 73% kcal, fat 10% kcal) or a 30% sucrose + 0.08% miglitol diet (S + M; protein 17% kcal, carbohydrate 73% kcal, fat 10% kcal, miglitol 0.08%) for eight weeks [20]. To examine phenotypes (tissue weight, tissue metabolites, plasma profile, mRNA levels), 18 week old male WT and KO mice were fed S or S + M diets for seven days. The diets were purchased from Research Diets Inc. (New Brunswick, NJ, USA). Miglitol was gifted by Sanwa Kagaku Kenkyuusho Co. (Nagoya, Japan). 

### 2.3. Liver Glycogen, Triglyceride, Cholesterol and Fructose Contents, and Plasma Profile Measurements

The liver glycogen content was measured as previously reported [12,19]. Liver lipids were extracted using the Bligh and Dyer method [21], and measured using triglyceride (Wako Pure Chemical Industries) and cholesterol E-tests (Wako Pure Chemical Industries). Liver fructose contents were measured by enzymatic methods [22]. Briefly, freeze-clamped tissues (100 mg) were homogenized in 2 mL of cold 6% perchloric acid, neutralized, and centrifuged. The assay is based on the oxidation of glucose as glucose-6-phosphate (G6P) using G6PDH. Fructose-6-phosphate is converted to G6P by the phosphoglucose isomerase enzyme, and subsequently oxidized by the G6PDH in the assay mixture. The fructose concentration is determined as the difference in G6P concentration before and after phosphoglucose isomerase treatment. All enzymes were purchased from Roche Custom Biotech Inc. Blood plasma was collected from the retro-orbital venous plexus following ad libitum feeding or after a 6-h fast. Blood glucose levels were measured using a FreeStyle Freedom monitoring system (Nipro, Osaka, Japan). Plasma triglycerides and total cholesterol levels were determined using the commercial kits, triglyceride E-test (Wako Pure Chemical Industries), and cholesterol E-test (Wako Pure Chemical Industries), respectively.

### 2.4. Cecal Contests Weight, Cecal Lactate Contents, and Intestinal Bacterial Flora

Mice fed with S or S + M were sacrificed at 19 weeks of age by cervical dislocation. After tissue weight, length of intestine and cecal contents were measured, the intestine and liver were immediately snap-frozen in liquid nitrogen and stored at −80 °C until further analysis of hepatic triacylglycerol and cholesterol contents, and quantitative PCR. For measurement of cecal lactate contents, frozen cecal content (20 mg) was homogenized in 80 μL of cold 6% perchloric acid, neutralized and centrifuged. Supernatants were collected and measured by a lactate measurement kit (Kyowa Medex). Terminal restriction fragment length polymorphism (T-RLFP) flora analysis of cecal contents was performed by Techno Suruga Labo Inc. (Shizuoka, Japan) [23]. 

### 2.5. Oral and Intraperitoneal Fructose-Loading Test

Fructose (3 g/kg BW) was orally or intraperitoneally injected into 14 weeks old male WT and KO mice. Plasma glucose was measured at the indicated times. For liver fructose contents and mRNA expression analyses, mice were sacrificed at 0, 1, 2 or 4 h, and the liver and intestine were removed and stored at −80 °C until further analysis.

### 2.6. RNA Isolation and Quantitative Real-Time PCR

Total RNA isolation, cDNA synthesis and real-time PCR analysis were performed as previously described [12,19]. Real-time PCR primers for mouse/rat *ChREBP*, liver type pyruvate kinase (*Pklr*), glucose transporter 2 (*Glut2*), fibroblast growth factor-21 (*Fgf-21*) and RNA polymerase II (*Pol2*) have been previously reported [19]. Primers used for *Glut5*, *Khk*, and *Si* were as follows: *Glut5* forward, 5′-CGGCTTCTCCACCTGCCTC-3′, *Glut5* reverse, 5′-CGTGTCCTATGACGTAGACAATGA-3′; *Khk-C* forward, 5′-GCTGACTTCAGGCAGAGG-3′, *Khk-C* reverse, 5′-CCTTCTCAAAGTCCTTAGCAG-3′; *Si* forward, 5′-TTGATATCCGGTCCACGGTTCT-3′, *Si* reverse, 5′-CAGGTGACATCCAGGTTGCATT-3′. All amplifications were performed in triplicate. The relative amounts of mRNA were calculated using the comparative CT method. *Pol2* expression was used as an internal control.

### 2.7. Statistical Analysis

All values are presented as means ± SD. Data were analyzed using Tukey’s test. A value of *p* < 0.05 was considered statistically significant. 

## 3. Results

### 3.1. ChREBP Knockout Mice Show Intolerance to Modest Amounts of Sucrose and Miglitol Diet

**Figure 1:**
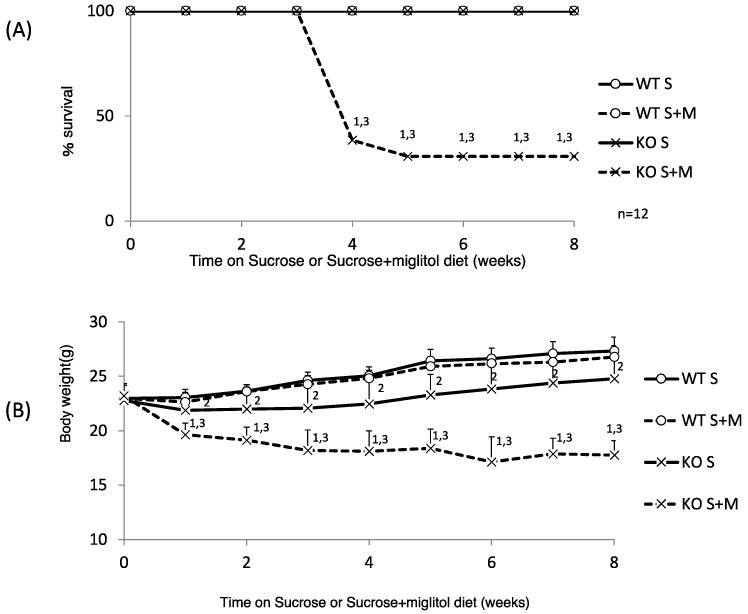
Thirty percent sucrose + 0.08% miglitol diet causes body weight loss and high lethality. Twelve week old male wild-type (WT) mice and *ChREBP* knockout (KO) mice were fed a 30% sucrose (S) or 30% sucrose plus 0.08% miglitol (S + M)-containing diet for eight weeks. (**A**) Survival rate. WT S, WT S + M, and K S, except KO S + M, survived. Data represented as % survival; (**B**) Body weight change. Data represented as mean ± SD (n = 12 per group). ^1^ KO S vs. KO S + M, *p* < 0.05, ^2^ WT S vs. KO S, *p* < 0.05, and ^3^ WT S + M vs. KO S + M, *p* < 0.05.

We have reported that a high-sucrose diet (60% sucrose) caused decreased appetite and eventual lethality in KO mice [12]. First, we investigated whether KO mice have any problems with sucrose digestion. We tested whether a medium amount of sucrose (30%) feeding caused body weight loss. A 30% sucrose diet was not lethal, although the body weight gain of 30% sucrose-diet-fed KO (KO S) mice was much lower than that of 30% sucrose-fed WT (WT S) mice (Figure 1A,B). 

Interestingly, the addition of miglitol, which inhibits sucrose digestion in the upper intestine, caused decreased body weight and increased mortality (75 and 75%, six and eight weeks after feeding the specific diet, respectively; Figure 1A,B). Next, we examined the following parameters one week after feeding the specific diet. The body weight changes and food intake of KO S mice were similar to those of WT S mice (Table 1). However, the body weight and food intake of sucrose plus miglitol (S + M) diet-fed KO (KO S + M) mice were significantly decreased compared with WT S + M mice. Consistently, the liver, epidydimal fat tissue and brown adipose tissue weight was decreased in KO S + M mice compared with WT S + M mice (Table 1). In contrast, the locomotor activity was similar among the groups (Table 1).

Regarding the plasma profile, the plasma glucose levels were lowest in KO S + M mice. Plasma triglyceride and total cholesterol levels in KO S and KO S + M mice were lower than those in WT S and WT S + M mice (Table 1). The liver triglyceride and cholesterol contents in KO S and KO S + M mice were also lower than those in WT S and WT S + M mice (Table 1). The liver glycogen content in KO S mice was increased; however, in KO S + M mice it was decreased owing to appetite loss (Table 1). Thus, KO S + M mice showed sucrose intolerance similar to high-sucrose diet-fed KO mice.

### 3.2. Sucrose Plus Miglitol Diet-Fed KO Mice Show Cecum Enlargement

**Figure 2:**
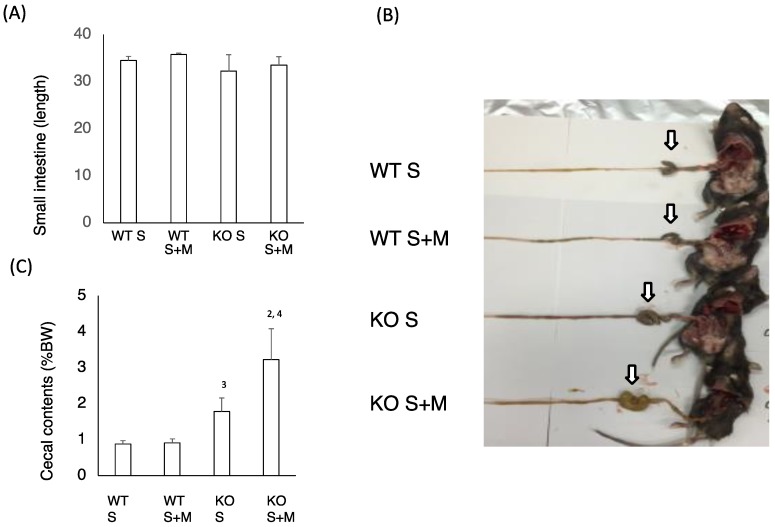

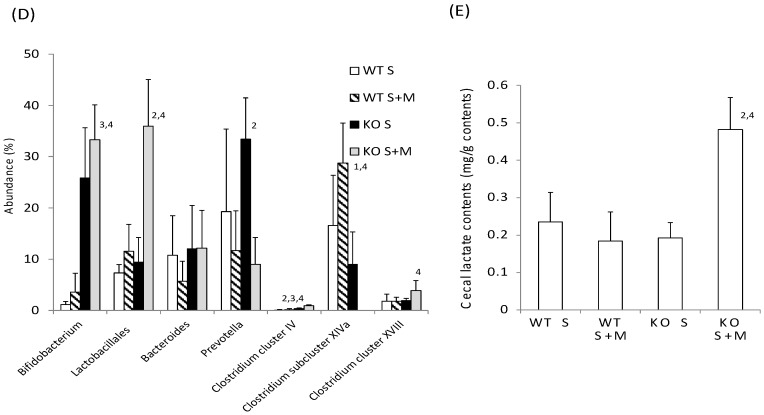
Sucrose plus miglitol diet-fed KO mice show cecal enlargement, higher lactate contents and altered intestinal flora. Eighteen week old male wild-type (WT) mice and *ChREBP*-knockout (KO) mice were fed a 30% sucrose (S) or 30% sucrose plus 0.08% miglitol (S + M)-containing diet for seven days. (**A**) Lengths (cm) of small intestine; (**B**) Representative image of intestinal enlargement; (**C**) Weight of cecal contents (% BW). Open arrows indcate cecum. Cecum in KO S and KO S + M were enlarged; (**D**) Gut microbes in cecum contents of WT and KO mice are expressed as a percentage of total DNA sequences; (**E**) Cecal lactate contents (mg/g). Data represented as mean ± SD (n = 6 per group). ^1^ WT S vs. WT S + M, *p* < 0.05, ^2^ KO S vs. KO S + M, *p* < 0.05, ^3^ WT S vs. KO S, *p* < 0.05, and ^4^ WT S + M vs. KO S + M, *p* < 0.05. BW: body weight.

Next, we checked the intestinal changes in WT and KO mice. The length of the small intestine was comparable in WT S, WT S + M, KO S and KO S + M mice (Figure 2A). The cecal enlargement and cecal contents in KO S mice were higher than those in WT S mice (Figure 2B,C). Although the food-loading test was performed only for one week, the cecal content in KO S + M mice was about 3.5 times higher than that in WT S and WT S + M mice (Figure 2B,C). Moreover, analysis of the intestinal flora and cecal contents showed that the ratios of *Bifidobacterium* and lactobacillales, and the cecal lactate contents were the highest in KO S + M mice (Figure 2D,E). In contrast, the abundance of clostridium cluster XIVa was dramatically diminished in KO S + M mice (Figure 2D).

### 3.3. Miglitol Affects the Expression of ChREBP Target Genes in the Intestine

**Figure 3:**
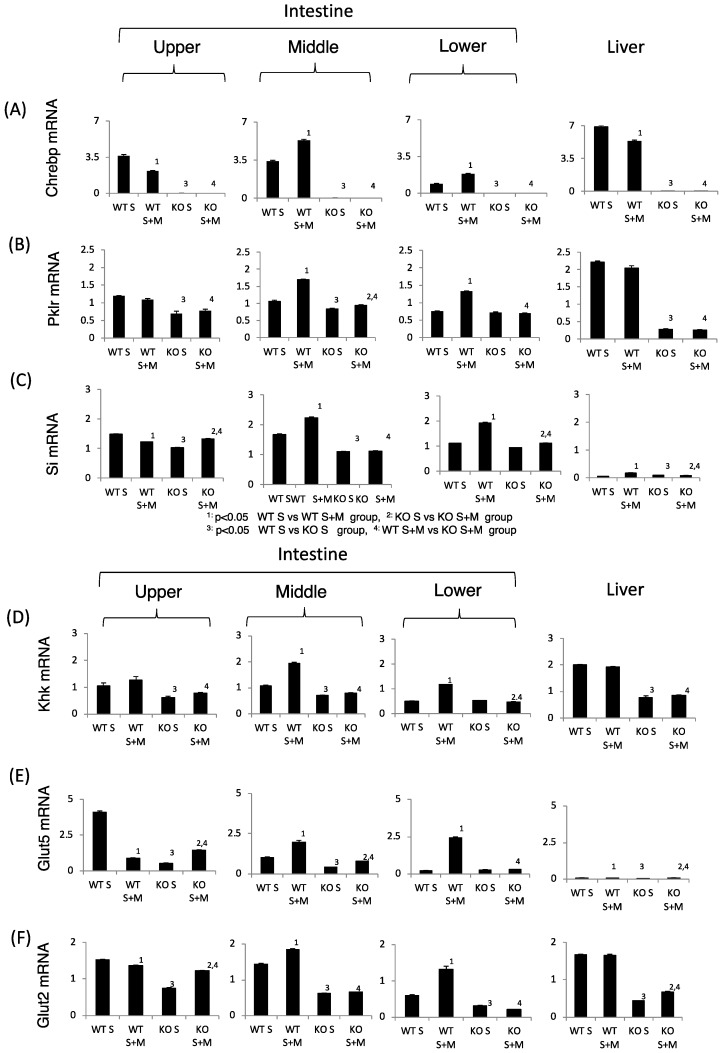
The effect of miglitol and the *ChREBP* gene deletion on genes related to ChREBP, fructose and sucrose metabolism. Eighteen week old male wild-type (WT) mice and *ChREBP*-knockout (KO) mice were fed a 30% sucrose (S) or 30% sucrose plus 0.08% miglitol (S + M)-containing diet for seven days. The intestine was divided into three parts (upper, middle and lower) and the mRNA levels were measured by real-time PCR. (**A**) *ChREBP*; (**B**) liver pyruvate kinase (*Pklr*); (**C**) sucrase isomerase (*Si*); (**D**) ketohexokinase (*Khk*); (**E**) glucose toransporter 5 (*Glut5*); and (**F**) glucose transporter 2 (*Glut2*). Data represented as mean ± SD (n = 3 per group). ^1^ WT S vs. WT S + M, *p* < 0.05, ^2^ KO S vs. KO S + M, *p* < 0.05, ^3^ WT S vs. KO S, *p* < 0.05, and ^4^ WT S + M vs. KO S + M, *p* < 0.05.

Next, we tested the sucrose and fructose metabolism in relation to gene expression. In WT S mice, the expression of sucrose metabolism (*Si*), fructose metabolism (*Glut2*, *Glut5* and *Khk*), and *ChREBP* and its target genes in the upper intestine were higher as compared with those in the lower intestine (Figure 3). Upon addition of miglitol, the mRNA expression of these genes was highest in the middle and lower intestine. In the liver, the mRNA expression of these genes was not affected by the addition of miglitol. Interestingly, the expression of *Glut5* mRNA in the liver was much lower than in the intestine (Figure 3E). By contrast, the mRNA levels of the abovementioned genes were lower in the KO mice than in the WT and the effect of miglitol on these mRNA levels was suppressed in KO S mice (Figure 3A–F). As compared with *Glut5* expression, *SGLT1* mRNA levels were not affected by *ChREBP* gene deletion (data not shown). Thus, we concluded that *ChREBP* regulates sucrose and fructose metabolism through gene expression.

### 3.4. Fructose Is Difficult to Metabolize in the Intestine, but Not in the Liver

**Figure 4:**
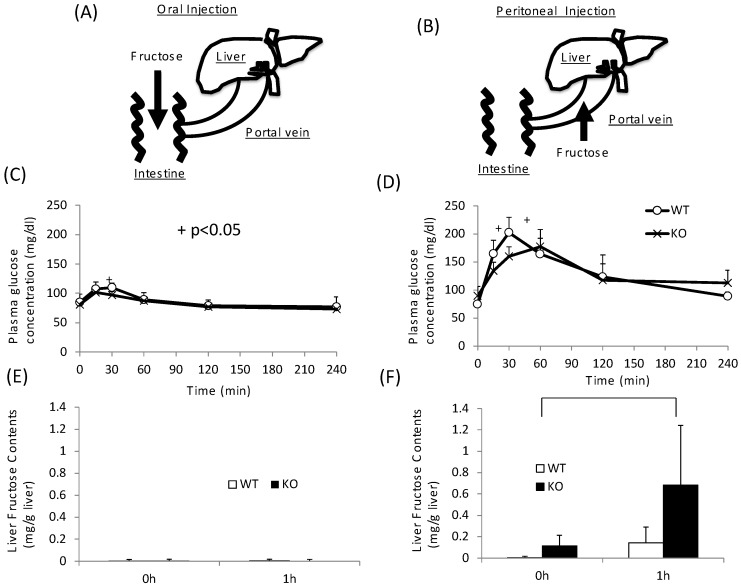
Oral and peritoneal fructose injection test. Oral (**A**) and perinoteal (**B**) injected fructose is abosorbed in intestine and portal vein, respectively. Time course of glucose concentration after oral (**C**) or peritoneal (**D**) fructose injection. Liver fructose content at 0 and 1 h after oral (**E**) or peritoneal (**F**) fructose injection. Data are presented as means ± SD (*n* = 6 per group). ^+^ WT vs. KO, *p* < 0.05.

As KO mice showed disturbance not only in sucrose metabolism but also in fructose metabolism, we next tested the role of intestinal and hepatic ChREBP in fructose metabolism. After oral fructose injection, fructose is absorbed in the intestine (Figure 4A). After peritoneal injection, fructose is absorbed in the portal vein (Figure 4B) [24]. In the oral fructose-loading test (3 g/kg BW), the plasma glucose levels in WT mice only modestly increased to 120 mg/dL at 30 min (Figure 4A). In KO mice, the plasma glucose levels at 30 min were slightly lower than those in WT mice (Figure 4A). By contrast, in peritoneal fructose loading, the plasma glucose levels in WT mice increased to 200 mg/dL at 30 min (Figure 4B). In KO mice, the plasma glucose levels were lower than those in WT mice, and the peak time shifted right (Figure 4B). Consistent with these results, the hepatic fructose content in the oral fructose-loading test (at 0 and 1 h) was undetectable (Figure 4C). Therefore, we concluded that fructose is difficult to metabolize and absorb in the intestine. In contrast, the fructose content after the peritoneal fructose-loading test at 1 h was measurable. Moreover, in KO mice, the hepatic fructose content at 1 h was about three times higher than that in WT mice (Figure 4D). These results suggest that hepatic fructose metabolism was inhibited at the level of KHK in the liver of KO mice.

### 3.5. ChREBP Regulates the Expression of Genes Related to Fructose Metabolism in the Intestine

**Figure 5:**
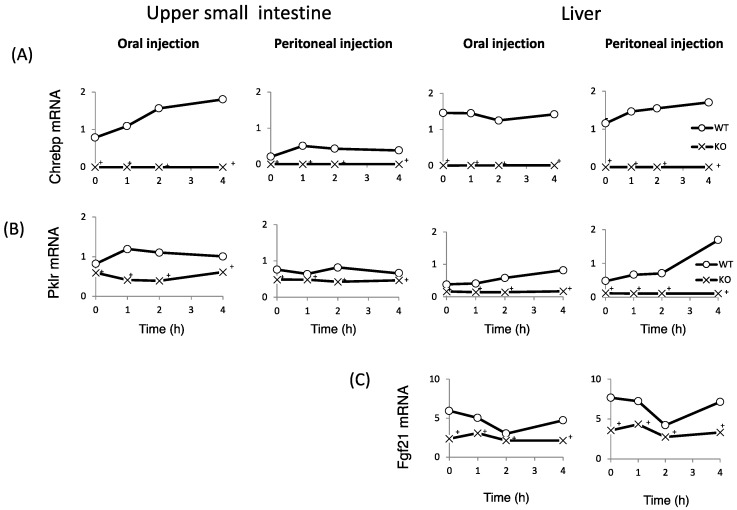

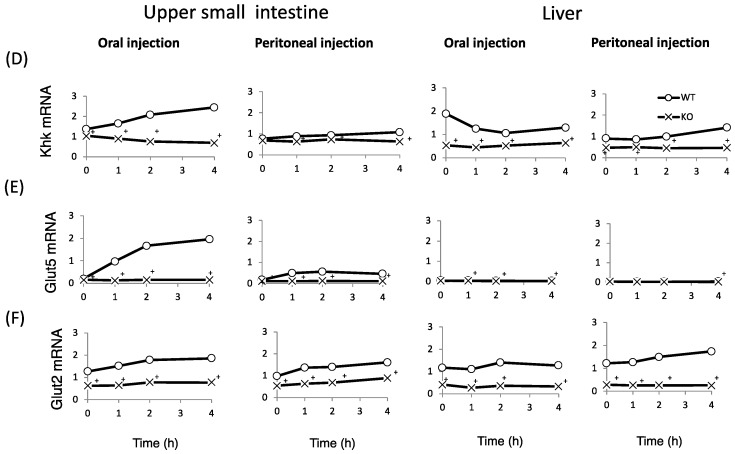
The effect of oral and peritoneal fructose injection on genes related to ChREBP and fructose metabolism. After oral or peritoneal fructose injection (3 kg/kg BW), the mRNA expression of *ChREBP* (**A**); liver type pyruvate kinase (*Pklr*) (**B**); fibroblast growth factor-21 (*Fgf21*) (**C**); ketohexokinase (*Khk*) (**D**); glucose transporter 5 (*Glut5*) (**E**); and glucose transporte 2 (*Glut2*) (**F**) in the intestine and liver was measured by real-time PCR analysis. *n* = 3 per group. ^+^WT vs. KO, *p* < 0.05.

Finally, we examined whether fructose induces the expression of intestinal and hepatic ChREBP target genes. After oral fructose injection, the expression of intestinal ChREBP target genes (*ChREBP*, *Pklr*) and fructose metabolism genes (*Glut2*, *Glut5*, and *Khk*) in WT mice increased in a time-dependent manner, while the mRNA expression of these genes was much lower in KO mice (Figure 5A–F). Consistent with the plasma glucose levels, the mRNA expression of the hepatic ChREBP target genes (*ChREBP*, *Pklr*, and *Fgf-21*) and fructose metabolism genes (*Glut2*, *Glut5*, and *Khk*) was not affected by fructose (Figure 5A–F). After peritoneal fructose injection, the hepatic mRNA expression of *ChREBP*, *Pklr*, *Glut2*, *Glut5*, and *Khk* in WT mice increased in a time-dependent manner; however, this induction was diminished in KO mice. By contrast, the intestinal mRNA levels of these genes were not affected by fructose injection (Figure 5A–F). In the liver, *Fgf-21* mRNA levels in KO mice were lower than those in WT mice. However, the hepatic *Fgf-21* mRNA levels in WT mice were not induced by oral or peritoneal fructose injection (Figure 5C). Thus, we concluded that oral and peritoneal fructose injection mainly induced intestinal and hepatic fructose metabolism genes regulated by ChREBP, respectively. 

## 4. Discussion

**Figure 6:**
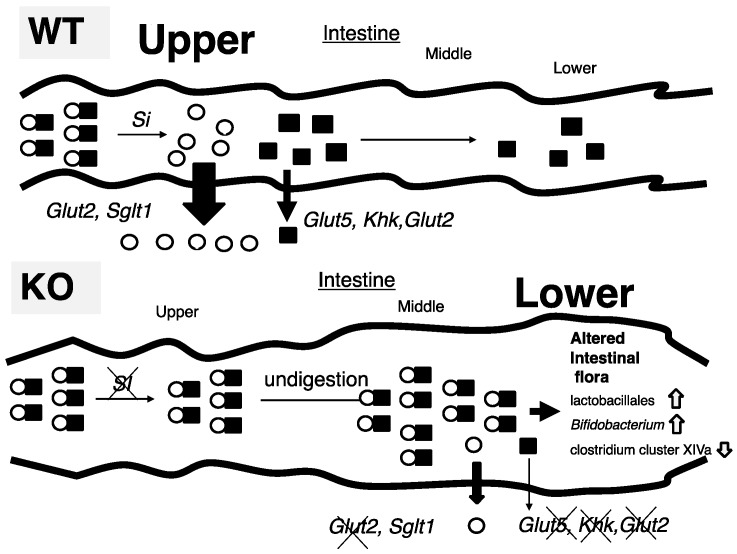
Schematic presentation of intestinal carbohydrate metabolism in wild-type and ChREBP knockout mice.

In this study, we tried to identify the mechanism by which *ChREBP*-KO mice show sucrose intolerance. Thirty percent sucrose (30%) diet-fed KO mice did not present the body weight loss and lethality seen in 60% sucrose diet-fed KO mice; however, Si inhibition by miglitol successfully exhibited sucrose intolerance. Increased fecal lactate contents, and increased growth of lactobacillales and *Bifidobacterium*, consistent with increased lactate contents, was seen only in S + M fed KO mice. These findings were consistent with decreased expression of sucrose and fructose metabolism-related genes, which are regulated by ChREBP. Moreover, oral and peritoneal fructose injection mainly induced ChREBP-regulated intestinal and hepatic fructose metabolism genes, respectively. These results suggest that alternations in the expression of both sucrose and fructose-related genes contribute to sucrose intolerance and fructose malabsorption in KO mice (Figure 6).

(A) In 30% sucrose plus 0.08% miglitol diet fed wild-type mice (WT), sucrose was digested into glucose and fructose in upper intestine. Glucose was almost absorbed in upper intestine. In contrast, fructose was partly absorbed and unabsorbed fructose was used for intestinal bacterial growth.
(B) In 30% sucrose plus 0.08% miglitol diet fed ChREBP knockout mice (KO), owing to decreased sucrase-isomaltase (SI) expression or SI inhibition by miglitol, undigested sucrose was moving into the lower intestine. Moreover, fructose absorption in KO was also decreased due to decreased intestinal glucose transporter 5 (*Glut5*), glucose transporter 2 (*Glut2*), and ketohexokianse (*Khk*) expression. Undigested sucrose and fructose in lower intestine and cecum affected intestinal bacterial flora (increased growth of lactobacillales and *Bifidobacterium* and decreased growth of clostridium cluster XIVa).

We have formerly reported that 60% sucrose diet-fed KO mice showed body weight loss and decreased food intake [12]. Despite the appetite loss, the cecum of dead 60% sucrose diet-fed KO mice was enlarged (unpublished data), hence, we wondered whether sucrose metabolism was disrupted in KO mice. As miglitol is a well-known Si inhibitor, the addition of miglitol caused an increased flux of undigested sucrose into the lower intestine. Consistent with these results, the addition of miglitol caused sucrose intolerance in KO mice fed a 30% sucrose diet, which, by itself, did not induce sucrose intolerance. Consistent with our hypothesis, KO S + M mice showed malabsorption (body weight, food intake, and diarrhea), similarly to the 60% sucrose diet-fed KO mice. Therefore, the increased flux of undigested sucrose into the lower intestine was partly due to the pathology of sucrose intolerance in KO mice.

S + M fed KO mice showed cecal enlargement in addition to body weight and appetite loss. Moreover, the ratios of lactobacillales and *Bifidobacterium* increased and the ratio of clostridium cluster XIVa reciprocally diminished in these mice. As the growth of these bacteria favors sucrose and fructose, these results suggest that undigested sucrose was moving into the lower intestine and cecum, and promoted the growth of lactobacillales and *Bifidobacterium* [25,26]. In contrast, the abundance of clostridium cluster XIVa increased in mice fed with high-fat diets [27]. Our data showed that Si inhibition did not change the gut microbiota in WT, which is consistent with the finding that Si inhibition by inulin-type fructans did not change the total number of bacteria in the cecal content and did not induce a bifidogenic effect [28]. However, the abundance of clostridium cluster XIVa was diminished in KO S + M mice. As these changes in KO mice were caused by a 60% sucrose diet and by an S + M diet, we concluded that sucrose intolerance was partly due to both Si suppression and a large amount of sucrose intake, resulting in an increased flux of undigested sucrose into the lower intestine. 

These phenotypes were similar to those of human SI deficiency patients [29]. After weaning from breast-feeding, human congenital SI deficiency patients experienced stomach cramps, bloating, excess gas production, and diarrhea, resulting in failure to gain weight and malnutrition. Most affected children have improved tolerance to sucrose and maltose as they get older. Moreover, α-glucosidase inhibitors (miglitol, voglibose, and acarbose) have gastrointestinal side effects such as flatulence, diarrhea, soft stool, and abdominal discomfort [30]. As S + M KO mice were sucrose-intolerant, KO mice may have another important metabolic defect, such as fructose malabsorption. 

Indeed, high-fructose diet-fed intestine-specific *ChREBP*-KO mice showed cecal enlargement and body weight loss similar to high-fructose diet-fed *GLUT5*^−/−^ mice, a model of fructose malabsorption [18,31]. These phenotypes appear similar to those of S + M KO mice. GLUT5 is mainly expressed in the intestine and kidneys, and much less in the liver [32]. Fructose absorption in mice and humans appears to be limited at high fructose concentrations, which is consistent with the limited absorption capacity of a facilitated transport system [33,34]. Moreover, in these *GLUT5*-KO mice, fructose absorption was decreased by 75% in the jejunum and the concentration of serum fructose was decreased by 90%, compared with WT mice [31]. Therefore, decreased “intestinal” *Glut5* mRNA may contribute to the lower intestinal fructose absorption in KO mice, suggesting that S + M-fed KO mice have not only sucrose intolerance, but also fructose malabsorption. From a clinical viewpoint, metformin sometimes causes abdominal discomfort (diarrhea and vomiting) [35]. Considering metformin can inhibit ChREBP activity [36], abdominal side effects may be due to suppression of ChREBP, and thereby decreased *Glut5* mRNA expression. If excess amounts of carbohydrates are consumed by patients with diabetes mellitus, the combination therapy of metformin and α-glucosidase inhibitor may increase abdominal side effects.

SI has important roles in the regulation of intestinal sucrose absorption [37]. SI is an enzyme that digests sucrose into glucose and fructose. *Si* mRNA is induced by sucrose and fructose [13,38]. Moreover, it has been reported that glucose “negatively” regulates human *Si* gene expression through two HNF binding sites in Caco-2 cells [39,40]. Therefore, it is reasonable that ChREBP does not directly regulate SI. However, we found that *Si* mRNA levels in the intestine of KO mice were lower than those in WT. We considered some potential pathways through which ChREBP indirectly regulates *Si* mRNA expression. First, the amount of sucrose intake by KO mice may be lower than the intake by WT because of appetite loss in KO mice. Second, intracellular metabolites derived from sucrose may be a signal for induction of SI genes. As ChREBP regulates glucose and fructose metabolism, intracellular metabolites may be decreased in KO mice. Interestingly, it has been reported that, independently of ChREBP, fructose uniquely induces *SREBP1c* and fatty acid synthesis genes, resulting in impaired insulin signaling [41]. Although further investigation is still needed, decreased *Si* mRNA levels in KO mice also partly contribute to the pathogenesis of sucrose intolerance.

In addition to decreased sucrose metabolism, decreased fructose metabolism has a more important role in the pathogenesis of sucrose intolerance in KO mice. We and other groups have reported that ChREBP has an important role in regulating fructose metabolism [11,12,14,15,16,17]. Many of the fructose metabolism genes (*Glut2*, *Glut5*, *Khk*, and *Aldob*) are ChREBP-target genes [12,17,18]. The mRNA levels of *Khk*, *Glut2* and *Glut5* in intestine-specific *ChREBP*-KO mice were much lower than in WT mice after oral fructose injection [18]. Consistently, our data showed that the mRNA levels of *Khk*, *Glut2*, and *Glut5* in KO mice were much lower than in WT mice. Moreover, oral fructose injection induced *Khk*, *Glut2*, and *Glut5* mRNA levels in a time-dependent manner only in WT mice. Moreover, intestinal KHK has important roles in intestinal fructose metabolism [7,42]. Low doses of fructose are ~90% cleared by the intestine and high doses of fructose (≥1 g/kg) overwhelm intestinal fructose absorption and clearance, resulting in fructose reaching both the liver and colonic microbiota [42]. Interestingly, Intestinal fructose clearance is augmented both by prior exposure to fructose and by feeding. These were compatible with our data. Intestinal *Khk* mRNA was induced by fructose and ChREBP gene deletion diminished *Khk* induction by fructose. Accordingly, these results reconfirmed that ChREBP coordinately regulates intestinal fructose metabolism by modulating *Khk*, *Glut2*, and *Glut5* gene expression.

Hepatic KHK has important roles in liver fructose metabolism [43,44]. It has been reported that the plasma fructose levels in *Khk*^−/−^ mice were 10 times higher than those in WT and *Glut5*^−/−^ mice [43]. Consistently, the hepatic fructose content in KO mice was much higher after peritoneal fructose injection, which is consistent with decreased *Khk* mRNA levels in the liver of KO mice. As with hepatic fructose transport, hepatic *Glut5* mRNA levels were much lower than in the intestine, which is consistent with a previous study [32]. Considering that the plasma fructose levels in *Glut5*^−/−^ mice were much lower than in *Khk*^−/−^ mice, other fructose transporters may regulate hepatic fructose uptake. Our data suggest that hepatic *Khk*, rather than *Glut5*, regulates hepatic fructose metabolism.

*Fgf-21* is induced by starvation through PPAR alpha activation [45]. Dietary protein restriction causes Fgf-21 induction through the amino acid sensor GCN2 activation [46]. Moreover, we formerly reported that Fgf-21 is regulated by ChREBP [47]. Fructose feeding increase plasma fructose levels [48]. In this study, hepatic *Fgf-21* mRNA levels in KO mice were much lower than those in WT mice, however, fructose induction of *Fgf-21* mRNA were not seen in both mice, which were not consistent with other reports. PPARα is also required for the ChREBP-induced glucose response of Fgf-21 regulation [49]. Moreover, glucagon and insulin cooperatively stimulate fibroblast growth factor 21 gene transcription by increasing the expression of activating transcription factor 4 [50]. Therefore, in vivo regulation of Fgf-21 expression is complicated. 

In this study, undigested excess fructose entered into the lower intestine, resulting in bacterial overgrowth. Fructose malabsorption causes irritable bowel syndrome. Moreover, excess fructose intake might increase colorectal cancer risk [51]. Interestingly, Aldolase B overexpression is associated with poor prognosis and promotes tumor progression by epithelial-mesenchymal transition in colorectal adenocarcinoma [52]. As Aldolase B is a ChREBP-target gene [53], colorectal ChREBP activation by undigested excess fructose might cause colorectal tumor progression. These suggested that intestinal fructose metabolism by ChREBP might be associated with irritable bowel syndrome and colorectal cancer.

## 5. Conclusions

In conclusion, both sucrose feeding and Si inhibitor caused sucrose intolerance and fructose malabsorption in *ChREBP*-KO mice. ChREBP coordinately regulates sucrose and fructose metabolism by modulating the mRNA expression of intestinal *Si* and *Glut5*, and hepatic *Khk*. Considering intestinal absorption of fructose is more difficult than that of glucose, intestinal ChREBP rather than hepatic ChREBP has an important role in the pathology of sucrose intolerance and fructose malabsorption.

## Figures and Tables

**Table 1:** The effect of 30% sucrose and 0.08% miglitol diet on wild-type mice and ChREBP knockout mice.

	WT S	WT S + M	KO S	KO S + M
BW (g) before	31.0 ± 1.77	29.8 ± 1.83	27.7 ± 1.62 ^(3)^	26.6 ± 1.21 ^(4)^
BW (g) after	29.3 ± 1.22	27.6 ± 0.91 ^(1)^	25.2 ± 1.07 ^(3)^	20.9 ± 1.00 ^(2)(4)^
BW (％) Difference	−5.38 ± 2.47	−7.5 ± 3.88	−8.97 ± 3.21	−21.5 ± 2.14 ^(2)(4)^
Liver (%BW)	5.33 ± 0.30	5.23 ± 0.23	7.12 ± 1.79 ^(3)^	4.97 ± 0.57 ^(2)^
Epidydimal Fat Weight (%BW)	1.78 ± 0.55	1.69 ± 0.32	1.35 ± 0.30 ^(3)^	0.47 ± 0.16 ^(2)(4)^
Brown Adipose Tissue (%BW)	0.40 ± 0.09	0.38 ± 0.05	0.30 ± 0.07 ^(3)^	0.26 ± 0.06 ^(4)^
Locomotor activity (counts/day)	14550 ± 3788	12778 ± 2984	12875 ± 2303	10800 ± 2066
Food Intake (g/day)	2.51 ± 0.63	2.33 ± 0.26	2.53 ± 0.17	1.77 ± 0.30 ^(2)(4)^
Plasma Glucose (mg/dL)	100.6 ± 9.6	96.3 ± 8.3	80.3 ± 10.8 ^(3)^	57.6 ± 6.8 ^(2)(4)^
Plasma Triglyceride (mg/dL)	137.2 ± 49.4	181.7 ± 54.2	72.7 ± 17.5	70.2 ± 14.2 ^(4)^
Plasma T-Chol (mg/dL)	127.5 ± 15.3	130.6 ± 4.4	60.3 ± 7.8	65.4 ± 6.46 ^(4)^
Liver Glycogen (mg/g liver)	38.6 ± 14.3	50.4 ± 17.4	83.5 ± 36.2 ^(3)^	56.9 ± 27.4
Liver Triglyceride (mg/g liver)	6.60 ± 1.97	5.54 ± 1.50	2.72 ± 0.84 ^(3)^	1.35 ± 0.45 ^(4)^
Liver Cholesterol (mg/g liver)	0.99 ± 0.32	1.54 ± 0.79	0.44 ± 0.14	0.56 ± 0.33 ^(4)^

Thirty percent sucrose fed wild-type mice (WT S), 30% sucrose plus 0.08% miglitol fed wild-type mice (WT S + M), 30% sucrose fed ChREBP knockout mice (KO S), and 30% sucrose plus 0.08% miglitol fed ChREBP knockout mice (KO S + M); BW: body weight; T-chol: total cholesterol. ^(1)^ WT S vs. WT S + M, *p* < 0.05, ^(2)^ KO S vs. KO S + M, *p* < 0.05, ^(3)^ WT S vs. KO S, *p* < 0.05, and ^(4)^ WT S + M vs. KO S + M, *p* < 0.05.
